# Birth weight and childhood obesity: effect modification by residence and household wealth

**DOI:** 10.1186/s12982-021-00096-2

**Published:** 2021-05-11

**Authors:** Helen Andriani

**Affiliations:** Department of Health Policy and Administration, Faculty of Public Health, Universitas Indonesia, Lingkar Kampus Raya Universitas Indonesia Street, Depok, 16424 Indonesia

**Keywords:** Low birth weight, Obesity, Urban–rural, Indonesia, Household wealth

## Abstract

**Background:**

There are both genetic and environmental factors which contribute to a child’s chances of being obese. When low birth weight (LBW) has been specifically evaluated relative to its association with childhood obesity, the results have produced conflicting findings. This study aims to describe the relationship between birth weight and childhood obesity and investigate the influence that residence and household wealth has on this relationship.

**Methods:**

I performed a secondary analysis on the 2013 Riskesdas (or Basic Health Research), a cross-sectional, nationally representative survey of the Indonesian population. Height, weight, information regarding child’s birth weight*,* and basic characteristics of the study population were collected from parents with children aged 0 to 5 years (n = 63,237) in 2013. The exposure was child’s birth weight and the outcomes were child’s current weight, BMI z-score, and obesity. Data were analyzed by using multiple linear regression and multiple logistic regression.

**Results:**

I found a significant increase in the weight, BMI z-score, and risk of childhood obesity to be associated with LBW. LBW children in rural area were associated with higher BMI z-score (mean ± standard error: 1.44 ± 0.02) and higher odds (odds ratio (95% confidence interval): 7.46 (6.77–8.23)) of obesity than those in urban area. LBW children from low class families were associated with higher BMI z-score (1.79 ± 0.04) and had higher odds (14.79 (12.47–17.54)) of obesity than those from middle class and wealthy families.

**Conclusions:**

Effective prevention and intervention to childhood obesity as early as possible are imperative. As far as this study was concerned, efforts, policies, and targets are required to reduce the prevalence of LBW. Children born of LBW, who live in a rural area and from low income families, should be emphatically intervened as early as possible.

**Supplementary Information:**

The online version contains supplementary material available at 10.1186/s12982-021-00096-2.

## Background

One of the most challenging public health problems of the twenty-first century is childhood obesity. In almost all countries, there has been a marked increase in childhood obesity prevalence over the past thirty to forty years [1]. Few studies have reported the relationship between obesity in children and risk factors for cardiovascular disease, dyslipidemia, insulin resistance, high blood pressure, left ventricular mass and endothelial function abnormalities in later life [2], which is affected by the behavior, genetics, and community of a person as it can influence the ability to make healthy choices.

Both genetic and environmental factors contributed to a child’s possibilities of being obese [3]. When low birth weight (LBW) has been specifically evaluated relative to its association with childhood obesity, the results have produced conflicting findings. Previous studies demonstrated that there was no association between obesity and LBW [4–6], while others showed that LBW (< 2500 g) was associated with a decreased risk of obesity [7–9]. LBW is associated with a lower risk of subsequent child overweight/obesity due to a more adverse body composition, e.g. increased central adiposity. Several studies have documented the positive association between LBW and later obesity in children [10–13]. LBW children will gain weight more rapidly in order to make up their lack of growth, which is called catch up growth [14]. LBW is a reflection of nutritional deprivation in uterus [15] and might impair the development of the fetal pancreas [16]. Consequently, it could lead to increased susceptibility to childhood obesity and non-communicable disease.

Differences in study design, study population or small sample size might lead to the differences in these conclusions. However, birth weight is not the individual cause. There are some other factors that may impact the relationship between birth weight and childhood overweight and obesity. Particularly, common characteristics in the prevalence of childhood overweight and obesity in Indonesia include the differences in rural/urban residence and higher prevalence of overweight and obesity amongst children from higher economic status family [17, 18]. However, studies comprehensively testing whether birth weight and childhood obesity differ by residence and household wealth are inadequate. The findings of this study contribute to fill the gap of the existing body of knowledge on the specific association and its conflicting findings between LBW and child obesity, provide information for policymakers, stakeholders, and decision-makers, as well as develop effective intervention strategies to prevent and control childhood obesity in vulnerable populations or areas. I aim to describe the association between birth weight and childhood obesity and examine the influence that residence and household wealth has on this relationship in children.

## Methods

### Data sources

This study involved a secondary analysis from the 2013 Riskesdas (or Basic Health Research) survey, a cross-sectional, nationally representative survey of the Indonesian population. The 2013 Riskesdas is the third survey conducted in Indonesia under the National Institute of Health Research and Development (NIHRD), Ministry of Health Republic of Indonesia*.* A two-stage, stratified cluster sampling approach was used for the selection of the survey sample. Two sampling frames were done in two stages (two stage sampling). In the first stage, implicit stratification of all Census Blocks (CB) from the 2010 Population Census (PC) was carried out based on the welfare strata. Based on the PC 2010 master frame there are 720,000 CB, then 180,000 CB (25%) are taken in a Probability Proportional to Size (PPS) to be the sampling frame for the selection of CB. Furthermore, a systematic selection of the number of CB in each strata of urban/rural per regency/city was made to produce a census block sample list. The total amount of CB selected is 30,000 CB. In the second stage, 10 households were selected in each CB. The frame for the household systematic sample selection is the updated list of ordinary households in the selected CBs, with the highest educational implicit stratification of the head of the household. Sampling was conducted among a national sample of 150 sub census blocks in all 33 provinces with the total 497 districts/cities in Indonesia. A complete interview was obtained for 294,959 households from targeted 300,000 households (98.3%). The eligible children included all biological, step, or adopted children of the household head and spouse, as well as any children fostered to any adult in the household.

### Measurement

The anthropometric measurements (height and weight) and information regarding child’s birth weight and basic characteristics of the study population were collected from parents with children aged 0 to 5 years in 2013. Standing height measures (for children over age two) and recumbent lengths (for younger children) were taken using “Multifunction”; measures of weight were taken using a digital weight scales "Fesco" brand, calibrated daily. Both of these measuring instruments have been used in survey work in other countries and are suitable for field work given their portability, durability, and accuracy [19].

Height and weight were used to calculate Body Mass Index (BMI). BMI z-scores were determined for each child based on the 2006 WHO Child Growth Standards for children under five years old, age and gender specific. Underweight was defined as BMI z-score ≤  − 2 SD. Normal weight was defined as − 2 SD < BMI z-score < 2 SD. Overweight was defined as 2 SD ≤ BMI z-score < 3 SD. Obese was defined as BMI z-score ≥ 3 SD [20, 21].

There were 82,666 children under five years old in 2013. Of those, a total of 11,009 (13.3%) children with missing data on height and weight had to be eliminated from the sample. Children classified as underweight (8420 children or 10.2%) according to WHO [20, 21] were also excluded, leaving normal weight, overweight, and obese status for the analysis. The final sample included 63,237 children. Child’s birth weight was based on self-reported maternal pregnancy history and birth outcomes. Mothers of study subjects were interviewed (i.e., How much did your baby weigh at birth (grams)?) In birth weight variable, LBW means a birth weight of < 2500 g, and normal birth weight was defined as a birth weight of ≥ 2500 g. There are three outcome variables: (1) child’s current weight (kg); (2) BMI z-score; (3) obesity (non-obesity vs obesity). “Non-obesity” group was a combination of normal weight and overweight, defined as -2 SD < BMI z-score < 3 SD, while “obesity” group was defined as BMI z-score ≥ 3 SD.

### Covariates

I considered the following as covariates for childhood obesity: child’s age (0, 1, 2, 3, and 4), child’s gender (boy and girl), breastfeeding (no and yes), mother’s education (none, elementary, junior high school, senior high school, and post-graduate), parental BMI (both parents < 25 kg/m^2^, only mother ≥ 25 kg/m^2^, only father ≥ 25 kg/m^2^, and both parents ≥ 25 kg/m^2^), household wealth (lowest (Q1), middle (Q2 + Q3), and highest (Q4 + Q5)), maternal and child health (available and not available), and residence (urban and rural). Household wealth in the 2013 Riskesdas was measured using a wealth index that constructed using Principle Component Analysis (PCA), generating quintiles ranging from quintile 1 (poorest) to quintile 5 (richest). The 12 variables used for PCA—source of drinking water, type of cooking fuel, use of toilet facilities, type of toilet, behaviour of stool disposal, type of light, motor cycle ownership, TV ownership, boiling water heater ownership, 12 kg gas cylinder ownership for cooking, refrigerator ownership, and car ownership—were based on the 2010 Indonesia National Socioeconomic Survey with some modification to existing economic status related variables in the 2013 Riskesdas. These covariates allowed for the control of variables that might influence childhood obesity. In addition to significant covariates, backward elimination was performed as the variable selection procedure to retain important confounding variables, resulting potentially in a slightly richer model. The overall model is also evaluated using the goodness of fit test by likelihood ratio test and Akaike’s Information Criterion (AIC).

### Statistical analyses

Data were analyzed by using chi-square, and multiple linear regression, and multiple logistic regression by adjusting the sampling weight for survey analysis. Chi-squared tests were used to test for differences between child’s birth weight for child’s current weight status and socio-economic status, area of residence and maternal and paternal characteristics of the study population. Using multivariable linear regression, controlling for covariates, I assessed the relationships of child’s birth weight with child’s weight and BMI z-score. A logistic regression model, with non-obesity as a reference category estimated the odds ratios (ORs) and 95% confidence intervals (CIs) of overweight. The statistical threshold for significance set at 0.05. All statistical analyses were performed by using SPSS 25.0 for Windows.

## Results

**Fig. 1:**
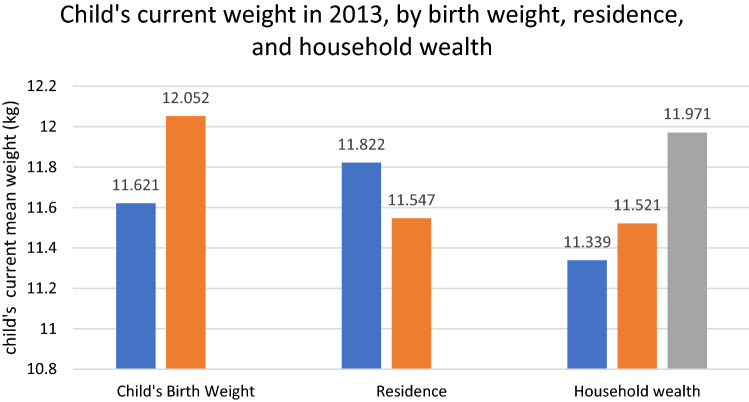
Child’s mean weight (kg), by birth weight (normal vs LBW), residence (urban vs rural), and household wealth (Q1 vs Q2 + Q3 vs Q4 + Q5)

A sample of 63,237 children had an average weight of 1167 kg (SD = 3.65) and an average BMI z-score of 0.25 (SD = 1.49). Figure 1 shows the distribution of child’s mean weight (kg), by birth weight, residence, and household wealth. Normal birth weight children had a mean weight of 11.62 kg (SD = 3.53) and LBW children had a mean weight of 12.05 kg (SD = 4.39). Urban children had mean weight of 11.82 kg (SD = 3.78), whereas rural children had mean weight of 11.55 kg (SD = 3.52). According to household wealth group, children from low-income family (Q1) had mean weight of 11.34 kg (SD = 3.39), children from middle class family (Q2 + Q3) had mean weight of 11.52 kg (SD = 3.49), whereas those from highest income group (Q4 + Q5) had mean weight of 11.97 kg (SD = 3.87).

The percentages of non-obese children and obese children were 93.3% and 6.7%, respectively. Breastfeeding, parental BMI, household wealth, maternal and child health, residence, and child’s weight status showed significant relationship with child’s birth weight. Around 88% and 12% children had normal and LBW, respectively. Children who were not breastfed (42.6%), children of parents both having a BMI ≥ 25 kg/m^2^ (21.8%), a poor family (13.1%), non-availability of maternal and child health (12.5%), lived in rural area (12.7%), and obese children (46.4%) were more likely to have LBW (Table 1). In both urban and rural areas, obese children were more likely to have LBW, with higher percentage of LBW among obese children in rural areas (53.0%) compared to those in urban areas (38.6%) (available at Additional file 1: Table S1). According to child’s weight status in each birth weight category stratified by household wealth category, obese children were more likely to have LBW, with higher percentage of LBW among obese children in Q1 or low-income family (66.9%) compared to those from middle class family (49.1%) and high-income family (35.8%) (available at Additional file 1: Table S2).

**Table 1:** Basic characteristics of the study population and child’s weight status across categories of child’s birth weight

	All	Child’s weight	Child’s birth weight		*p*
	N = 63,237 (100%)	Mean (SD)	Normal n (%) (N = 55,735)	Low n (%) (N = 7489)	
Child’s age					
0	10,643 (16.8)	6.85 (1.88)	9239 (86.8)	1404 (13.2)	< 0.001
1	11,986 (19.0)	9.72 (1.64)	10,096 (84.2)	1889 (15.8)	
2	12,108 (19.1)	11.69 (2.06)	10,819 (89.4)	1286 (10.6)	
3	13,665 (21.6)	13.53 (2.45)	12,302 (90.1)	1357 (9.9)	
4	14,835 (23.5)	15.30 (2.92)	13,279 (89.5)	1553 (10.5)	
Child’s gender					
Boys	31,630 (45.6)	12.09 (3.69)	27,875 (88.1)	3755 (11.9)	0.837
Girls	31,597 (54.4)	11.53 (3.70)	27,860 (88.2)	3734 (11.8)	
Breastfeeding					
No	3580 (5.7)	11.70 (4.56)	2055 (57.4)	1525 (42.6)	< 0.001
Yes	59,657 (94.3)	11.82 (3.64)	53,680 (90.0)	5964 (10.0)	
Mother’s education					
None	5820 (10.1)	11.70 (3.62)	5088 (87.4)	731 (12.6)	0.140
Elementary	13,336 (23.2)	11.70 (3.54)	11,776 (88.3)	1558 (11.7)	
Junior high	13,288 (23.1)	11.79 (3.67)	11,659 (87.8)	1626 (12.2)	
Senior high	17,536 (30.4)	11.90 (3.80)	15,495 (88.4)	2014 (11.6)	
Post-graduate	7613 (13.2)	11.95 (3.87)	6736 (88.5)	877 (11.5)	
Parental BMI					
Both parents < 25 kg/m^2^	26,326 (55.9)	11.71 (3.60)	23,117 (87.8)	3202 (12.2)	< 0.001
Only mother ≥ 25 kg/m^2^	9821 (20.8)	11.88 (3.70)	8453 (86.1)	1365 (13.9)	
Only father ≥ 25 kg/m^2^	5966 (12.7)	11.94 (3.85)	5150 (86.3)	816 (13.7)	
Both parents ≥ 25 kg/m^2^	5009 (10.6)	12.11 (4.09)	3919 (78.2)	1090 (21.8)	
Household wealth					
Q1 (lowest)	12,620 (20.0)	11.34 (3.39)	10,968 (86.9)	1650 (13.1)	< 0.001
Q2 + Q3	24,175 (38.2)	11.52 (3.49)	21,384 (88.5)	2786 (11.5)	
Q4 + Q5 (highest)	26,442 (41.8)	11.97 (3.87)	23,383 (88.5)	3053 (11.5)	
Maternal and child health					
Available	44,597 (70.5)	11.74 (3.68)	39,431 (88.4)	5155 (11.6)	0.001
Not available	18,640 (29.5)	11.99 (3.76)	16,304 (87.5)	2334 (12.5)	
Residence					
Urban	28,809 (45.6)	11.82 (3.78)	25,694 (89.2)	3108 (10.8)	< 0.001
Rural	34,428 (54.4)	11.55 (3.52)	30,041 (87.3)	4381 (12.7)	
Child’s weight status					
Non-obese (normal + overweight)	59,026 (93.3)	11.54 (3.49)	53,481 (90.6)	5536 (9.4)	< 0.001
Obese	4211 (6.7)	13.50 (5.10)	2254 (53.6)	1953 (46.4)	
Child’s birth weight					
Normal (≥ 2500 g)	55,735 (88.2)	11.62 (3.53)	–	–	
Low (< 2500 g)	7489 (11.8)	12.05 (4.39)	–	–	

Breastfeeding, parental BMI, and household wealth were significantly associated with child’s birth weight. I found the mean weight and mean BMI z-score to be significantly associated with LBW. After adjusting for the covariates, LBW children had a mean weight of 0.79 kg (*p* < 0.001) and a mean BMI z-score of 1.17 (*p* < 0.001) higher than that of normal birth weight children. LBW significantly raised the odds of being obese (OR = 8.37, *p* < 0.01). After adjusting for the covariates, compared to normal birth weight, LBW showed a significant 6.29-fold increase in the odds for obesity (adjusted OR = 6.29, 95% CI [5.84, 6.78], *p* < 0.001) (Table 2).

**Table 2:** Mean weight and mean BMI z-score for obesity compared to non-obesity

		Linear regression				Logistic regression			
		weight^a^		BMI z-score^a^		obesity^ba^			
	n	mean ± SE	*p*	mean ± SE	*p*	cOR	*p*	aOR (95% CI)	*p*
Child’s birth weight									
Normal (≥ 2500 g)	55,735	11.71± 0.01	*–*	0.20 ± 0.01	*–*	1		1	
Low (< 2500 g)	7489	12.50 ± 0.03	< 0.001	1.37 ± 0.02	< 0.001	8.37	< 0.001	6.209 (5.84–6.78)	< 0.001

*cOR* crude odds ratio, *aOR* adjusted odds ratio, *CI* confidence interval

^a^adjusted for child’s age, child’s gender, breastfeeding, mother’s education, parental BMI, and household wealth, Maternal and Child Health, and residence

^b^adjusted for child’s age, child’s gender, breastfeeding, parental BMI, household wealth, and Maternal and Child Health

Adjusting for area of residence reduced birthweight by 151 g and this change was variable between groups. From the stratified analyses, after adjusting for the covariates, mean weight and mean BMI z-score was significantly associated with LBW in urban and rural areas. The mean weight of LBW children was 0.82 kg higher (*p* < 0.001) in urban area and 0.77 kg higher (*p* < 0.001) in rural area than that of normal birth weight children. Compared to normal birth weight children, the mean BMI z-score of LBW children was 1.02 higher (*p* < 0.001) in urban area and 1.27 higher (*p* < 0.001) in rural area. Compared to normal birth weight children, the odds ratio of being obese was statistically significantly higher among LBW children in urban area (OR = 6.50, *p* < 0.01) and in urban area (OR = 10.33, *p* < 0.01). After adjusting for the covariates, the odds ratio for obesity of LBW children in urban and rural area was 4.76 (95% CI [4.25, 5.33], *p* < 0.01) and 7.46 (95% CI [6.77, 8.23], *p* < 0.01), respectively (Table 3).

**Table 3:** Mean weight and mean BMI z-score for obesity compared to non-obesity, stratified by residence

	n	Urban (N = 28,809)							
		Linear regression				Logistic regression			
		Weight^a^		BMI z-score^a^		Obesity^ba^			
		mean ± SE	*p*	mean ± SE	*p*	cOR	*p*	aOR (95% CI)	*p*
Child’s birth weight									
Normal (≥ 2500 g)	32,749	11.87 ± 0.02	*–*	0.24 ± 0.01	*–*	1		1	
Low (< 2500 g)	3390	12.69 ± 0.05	< 0.001	1.26 ± 0.03	< 0.001	6.50	< 0.001	4.76 (4.25–5.33)	< 0.001

	n	Rural (N = 34,428)							
		Linear regression				Logistic regression			
		Weight^a^		BMI z-score^a^		Obesity^ba^			
		mean ± SE	*p*	mean ± SE	*p*	cOR	*p*	aOR (95% CI)	*p*
Child’s birth weight									
Normal (≥ 2500 g)	39,096	11.57 ± 0.02	*–*	0.17 ± 0.01	*–*	1		1	
Low (< 2500 g)	4676	12.34 ± 0.04	< 0.001	1.44 ± 0.02	< 0.001	10.33	< 0.001	7.46 (6.77–8.23)	< 0.001

*cOR* crude odds ratio, *aOR* adjusted odds ratio, *CI* confidence interval

^a^adjusted for child’s age, child’s gender, breastfeeding, mother’s education, parental BMI, household wealth, and Maternal and Child Health

^b^adjusted for child’s age, child’s gender, breastfeeding, parental BMI, household wealth, and Maternal and Child Health^a^adjusted for breastfeeding, parental BMI, and household wealth

On further adjustment with household wealth, birth weight was further reduced by 117 g and this change in LBW was varied among the household wealth groups of low, middle and wealthy. From the stratified analyses, after adjusting for the covariates, mean weight and mean BMI z-score was significantly associated with LBW in low, middle class, and wealthy families. The mean weight of LBW children was 1.04 kg higher (*p* < 0.001) in a low class family, 0.72 kg higher (*p* < 0.001) in a middle class family, and 0.70 kg higher (*p* < 0.001) in a wealthy family than that of normal birth weight children. Compared to normal birth weight children, the mean BMI z-score of LBW children was 1.74 higher (*p* < 0.001) in a low class family, 1.17 higher (*p* < 0.001) in a middle class family, and 0.88 higher (*p* < 0.001) in a wealthy family. Compared to normal birth weight children, the odds ratio of being obese was statistically significantly higher among LBW children of a low class family (OR = 19.31, *p* < 0.01), a middle class family (OR = 9.73, *p* < 0.01) and a wealthy family (OR = 5.21, *p* < 0.01). After adjusting for the covariates, LBW children of a low class family (adjusted OR = 14.79, 95% CI [12.47, 17.54], *p* < 0.01), a middle class family (adjusted OR = 6.72, 95% CI [5.94, 7.59], *p* < 0.01) and a wealthy family (adjusted OR = 4.01, 95% CI [3.58, 4.48], *p* < 0.01) were associated with higher odds of being obese (Table 4).

**Table 4:** Mean weight and mean BMI z-score for obesity compared to non-obesity, stratified by household wealth

	n	Q1 (N = 12,620)							
		Linear regression				Logistic regression			
		Weight^a^		BMI z-score^a^		Obesity^ba^			
		mean ± SE	*p*	mean ± SE	*p*	cOR	*p*	aOR (95% CI)	*p*
Child’s birth weight									
Normal (≥ 2500 g)	14,675	11.32 ± 0.03	*–*	0.05 ± 0.02	*–*	1		1	
Low (< 2500 g)	1748	12.36 ± 0.06	< 0.001	1.79 ± 0.04	< 0.001	19.31	< 0.001	14.79 (12.47–17.54)	< 0.001

	n	Q2 + Q3 (N = 24,175)							
		Linear regression				Logistic regression			
		Weight^a^		BMI z-score^a^		Obesity^ba^			
		mean ± SE	*p*	mean ± SE	*p*	cOR	*p*	aOR (95% CI)	*p*
Child’s birth weight									
Normal (≥ 2500 g)	27,288	11.55 ± 0.02	*–*	0.17 ± 0.01	*–*	1		1	< 0.001
Low (< 2500 g)	3028	12.27 ± 0.05	< 0.001	1.33 ± 0.03	< 0.001	9.73	< 0.001	6.72 (5.94–7.59)	

	n	Q4 + Q5 (N = 26,442)							
		Linear regression				Logistic regression			
		Weight^a^		BMI z-score^a^		Obesity^ba^			
		mean ± SE	*p*	mean ± SE	*p*	cOR	*p*	aOR (95% CI)	*p*
Child’s birth weight									
Normal (≥ 2500 g)	29,882	12.03 ± 0.02	*–*	0.30 ± 0.01	*–*	1		1	
Low (< 2500 g)	3290	12.73 ± 0.05	< 0.001	1.18 ± 0.03	< 0.001	5.21	< 0.001	4.01 (3.58–4.48)	< 0.001

*cOR* crude odds ratio, *aOR* adjusted odds ratio, *CI* confidence interval

^a^adjusted for child’s age, child’s gender, breastfeeding, mother’s education, parental BMI, Maternal and Child Health, and residence

^b^adjusted for child’s age, child’s gender, breastfeeding, parental BMI, and Maternal and Child Health^a^adjusted for breastfeeding and parental BMI

## Discussion

Obesity has become a worldwide epidemic not only in adults but also in children. I indicated representative population-based data concerning obesity in children in Indonesia. Approximately, 93.3% of children under five were non-obese, and 6.7% were obese. This study showed that the average weight and BMI z-score of LBW children was higher than the average weight and BMI z-score of children with normal birth weight. After adjusting for the covariates, compared to those with normal birth weight, children born to LBW were nearly six times higher estimated risk to become obese. LBW babies, who have early rapid “catch-up” growth in their first two years, had a higher levels of growth hormone, were more exposed to abdominal obesity, and were fatter than other children by age five [22]. “Deprived of nutrition” before birth may be begun for accelerated growth after birth, especially when the babies were exposed to a rich nutrient environment, including infant formula feeding. There were three primary physiological mechanisms as the mediators of the impacts of LBW in the later obesity development and other conditions. The principal system is the adjustment in the phenotype expression produced by the lack of replication of cells, which may lead the body to store energy as a versatile reaction. A second mechanism is a change produced in metabolism through hormone expression, introducing an affiliation between higher resistance to insulin and LBW. Another hypothesis is that LBW inclines people to be increasingly defenseless to environmental impacts present in posterior periods of the life cycle [23]. According to a study performed by Sawaya [24], catch-up growth in the recovery of LBW leads to higher fat mass and lower protein reserves in muscles. LBW children had less fat-free mass during youth and pre-adulthood [25]. These confirmations showed that LBW does not impact the event of overweight/obesity straightforwardly; however, it results in the body adjustment systems, such as catch-up growth and hormone variations, which could lead to overweight/obesity. That is why the assumption of the relationship between LBW and overweight/obesity needs to be continuously studied, particularly evaluating the body composition regarding fat-free mass and fat mass.

The findings from this study were in contrast with those reported by other studies. Most studies using odds ratios reported a positive association between birth weight and overweight/obesity in children, while the negative association between birth weight and subsequent obesity were demonstrated through weight gain, abdominal fat, central fat accumulation, body composition, and insulin resistance approach. There were few reports upon the relationship of LBW with obesity in later life, when in fact, it was usually presented separately. Many cohort studies have shown that after adjusting for age, sex, and birth weight, fast weight gain in infancy is related to an increased risk of consequent obesity among all children [26–28]. During the first year of life, weight gain (kg) was significantly correlated with subsequent childhood obesity (OR 1.46, 95% CI 1.27–1.67) and obesity (1.59, 1.29–1.97) [12]. LBW’s transition to a normal or increased BMI has been associated with changes in body composition during childhood: LBW was associated with increased central fat accumulation in children [29]. Studies from Australia have also reported the negative association between LBW and increased abdominal fat over a normal birth weight range (beta = − 0.18; 95% CI = − 0.31 to− 0.04, *p* = 0.009) [30]. A study in China revealed that LBW was associated with an increased risk of severe obesity (OR 1.27, 95% CI 1.03 to 1.55) but was not associated with obesity (OR 0.85, 95% CI 0.67 to 1.06) [13]. These studies suggest that birth weight can play a role in the early characterization of a risk group for overweight and obesity development in childhood.

Other studies showed that there was a positive association between birth weight, BMI *z*-scores, and increased odds of obesity among children, particularly those from high-income countries [6, 31–33]. The OR (1.79 (95% CI 1.11–2.89)) of childhood obesity started to rise after 4000 g of birth weight compared to the 2500–2999 g of birth weight group [34]. A study used fourteen rounds of the Health Survey for England between 2000 and 2014 showed that the association between birth weight and subsequent obesity was significantly more pronounced in children from low-income families, compared with children from high-income families. The differences between developed and developing countries in maternal weight and nutritional status before and during pregnancy and infant feeding could result in a varying degree of association between high birth weight and the risk of childhood obesity among these countries. An alternative theory may be that parents with low incomes may be more likely to lack the psychological and financial support to cope with children in high need [35].

This study confirms different mean weight, mean BMI z-score, and obesity risk of LBW children by different residences and household wealth. There was effect modification by the residence and household wealth. The mean weight of LBW children in the urban areas and wealthy families was higher than that of LBW children in the rural areas, middle class, and low-class families. However, the mean BMI z-score, odds ratio, and adjusted odds ratio for obesity of LBW children in rural areas and low-class families were higher than those of LBW children in urban areas, middle class, and wealthy families. Hulme and Blegen found that women living in rural areas had an increased risk of having LBW infants [36]. In the rural areas, individuals are generally having a homogenous profession and relying upon agriculture. The impacts of work on income were valuable; however, mothers who do their job in exhausting occupations, such as prolonged standing, are at risk for having LBW babies [37]. Additionally, rural women were underserved by prenatal and obstetric care that can avoid pregnancy complications [38]. Low socioeconomic status among women correlates with an increased risk for delivering LBW babies [39]. Research has demonstrated that at the regional and household level, poverty predicts LBW [40, 41]. Low-income families do not consume the suggested amounts of fruit, vegetables, meats, whole grains, and low-fat dairy items. They tend to consume a less nutritious diet. Mothers of a low-income family regularly want to improve their economy by working.

Few strengths of this study including a large sample size and considering a unique association between birth weight and child’s weight, BMI z-score, and childhood obesity in urban–rural settings and at different household wealth, especially in Indonesia. In this study, the number of participants was more extensive than those of other studies. The measurement methods measuring height and weight with trained interviewers (usually nurses) are more accurate than self-reported measurements. Nevertheless, the study highlights several limitations; The retrospective bias was unavoidable as one of the drawbacks of observational studies. Retrospective bias or retrospective impact bias is a tendency to believe that we have predicted the outcome of an event accurately, once the result is known. It reduces the ability to learn from the past (overestimating the impact of past events) and correct their forecasts. The association between birth weight and child weight status may be confounded by several potential factors, such as a family history of obesity-related disease, dietary energy intake, infant feeding advice including formula feeding, parental feeding behaviors, as well as more active play and sedentary behavior limitation, etc. [42, 43]. Those variables weren’t available and it is the main shortcoming of this research. As a measure of weight status, BMI might present misclassification problems that result in an estimation bias for the relationship between birth weight and childhood obesity. The child’s birth weight was based on self-reported maternal pregnancy history and birth outcomes, and it was not confirmed by tracking the detailed birth information of these enrolled children in their birth hospitals. Weight and BMI changes dramatically in the first five years of age. The association between birth weight and current weight by age might be worth exploring.

## Conclusion

Obesity was dramatically spreading among children under five in Indonesia. In this study, residence and household wealth were found to be an effect modifier. It is imperative to do powerful prevention and intervention to obesity in children as early as possible. Early risk factors identification was critical. A significant increase in weight and BMI z-score and an increased risk of childhood obesity were found to be associated with LBW. So far as this study was concerned, efforts, policies, and targets are required to reduce LBW prevalence. Children born of LBW, who live in a rural area and from low-income families, should be emphatically intervened as early as possible.

## Supplementary Information

**Additional file1**: **Table S1** Child’s weight status across categories of child’s birth weight, stratified by residence. **Table S2** Child’s weight status across categories of child’s birth weight, stratified by household wealth.

## Data Availability

All relevant data provided within the paper were obtained and generated from the Basic Health Research (Riskesdas) survey which is not publicly available. To apply for the use of such data, the applicant is required to comply with the terms and procedures set by The National Institute of Health Research and Development (NIHRD), Ministry of Health, Republic of Indonesia.

## Abbreviations

LBW: Low birth weight
NIHRD: The national institute of health research and development
BMI: Body mass index
OR: Odds ratio
