# Supplementary material for: Birth weight and childhood obesity: effect modification by residence and household wealth
Source: Emerg Themes Epidemiol. 2021 May 11;18:6. doi: 10.1186/s12982-021-00096-2 (PMC8111737; doi:10.1186/s12982-021-00096-2)
Supplement: Supplementary file 1 — Additional file1: Table S1 Child’s weight status across categories of child’s birth weight, stratified by residence. Table S2 Child’s weight status across categories of child’s birth weight, stratified by household wealth. [file 12982_2021_96_MOESM1_ESM.docx]

**Table S1** Child’s weight status across categories of child’s birth weight, stratified by residence

|  | **Urban (N = 28,809)** | | |
| --- | --- | --- | --- |
|  | **Child’s Birth Weight** | | ***p*** |
|  | **Normal**  **n (%)**  **(N = 25,694)** | **Low**  **n (%)**  **(N = 3108)** |  |
| Child’s Weight Status  Non-obese (Normal + Overweight)  Obese | 24,513 (91.2)  1181 (61.4) | 2367 (8.8)  741 (38.6) | <0.001 |
|  | **Rural (N = 34,428)** | | |
|  | **Child’s Birth Weight** | | ***p*** |
|  | **Normal**  **n (%)**  **(N = 30,041)** | **Low**  **n (%)**  **(N = 4381)** |  |
| Child’s Weight Status  Non-obese (Normal + Overweight)  Obese | 28,968 (90.1)  1073 (47.0) | 3169 (9.9)  1212 (53.0) | <0.001 |

Table S1 shows that in both urban and rural areas, obese children were more likely to have LBW, with higher percentage of LBW among obese children in rural areas (53.0%) compared to those in urban areas (38.6%).

**Table S2** Child’s weight status across categories of child’s birth weight, stratified by household wealth

|  | **Q1 (N = 12,620)** | | |
| --- | --- | --- | --- |
|  | **Child’s Birth Weight** | | ***p*** |
|  | **Normal**  **n (%)**  **(N = 10,968)** | **Low**  **n (%)**  **(N = 1650)** |  |
| Child’s Weight Status  Non-obese (Normal + Overweight)  Obese | 10,707 (90.5)  261 (33.1) | 1122 (9.5)  528 (66.9) | <0.001 |
|  | **Q2 + Q3 (N = 24,175)** | | |
|  | **Child’s Birth Weight** | | ***p*** |
|  | **Normal**  **n (%)**  **(N = 21,384)** | **Low**  **n (%)**  **(N = 2786)** |  |
| Child’s Weight Status  Non-obese (Normal + Overweight)  Obese | 20,611 (91.0)  773 (50.9) | 2041 (9.0)  745 (49.1) | <0.001 |
|  | **Q4 + Q5 (N = 26,442)** | | |
|  | **Child’s Birth Weight** | | ***p*** |
|  | **Normal**  **n (%)**  **(N = 23,383)** | **Low**  **n (%)**  **(N = 3053)** |  |
| Child’s Weight Status  Non-obese (Normal + Overweight)  Obese | 22,163 (90.3)  1220 (64.2) | 2373 (9.7)  680 (35.8) | <0.001 |

Child’s weight status in each birth weight category stratified by household wealth category in Table S2 shows that obese children were more likely to have LBW, with higher percentage of LBW among obese children in Q1 or low-income family (66.9%) compared to those from middle class family (49.1%) and high-income family (35.8%).
